# How does iliosacral bone tumor resection without reconstruction affect the ipsilateral hip joint?

**DOI:** 10.1186/s12891-018-2023-9

**Published:** 2018-04-04

**Authors:** Tao JIN, Weifeng LIU, Hairong XU, Yuan LI, Lin HAO, Xiaohui NIU

**Affiliations:** Department of Orthopedic Oncology Surgery, Beijing Ji Shui Tan Hospital, Peking University, No 31, Xin Jie Kou east street, Xi Cheng District, Beijing, 100035 People’s Republic of China

**Keywords:** Iliosacral bone tumor, No reconstruction, Hip joint, Coverage, Function

## Abstract

**Background:**

Whether reconstruction is more beneficial after iliosacral bone tumor resection remains controversial. Because of high rates of complications and recurrence, few patients benefit from reconstruction. The aim of this study is to assess functional outcomes and to reveal changes in the ipsilateral hip joint after partial iliosacral resection.

**Methods:**

From 1998 to 2016, 21 patients aged 20–66 years underwent iliosacral resection, 18 without reconstruction (group 1) and 3 with reconstruction (group 2). Function was evaluated using the Musculoskeletal Tumor Society 1993 rating scale (MSTS 1993), and disability was measured using the Toronto Extremity Salvage Score (TESS). I-A distance was defined as the distance from the iliosacral joint to the upper line of the acetabulum along the curved line. Group 1 were subdivided into two groups: group 1A included the patients with a defect less than one-third of the I-A distance and group 1B the remainder. Acetabulum-head index (AHI) and center-edge angle (CE angle) were measured. The relationship between defect length and femoral head coverage was analyzed.

**Results:**

The mean follow-up was 67.3 months. Eighteen patients were included in group 1 and three in group 2. Preoperative data of the 3 groups were statistically equivalent. In addition, no difference of postoperative functional outcome has been highlighted. The final average MSTS 1993 score was 93.6% in group 1 and 93.3% in group 2. The mean TESS was 98 in group 1 and 98.5 in group 2. AHI and CE angle between groups 1 and 2 were not different. The AHI was 80 ± 5.4% in group 1A and 67 ± 9.0% in group 1B (*t* = − 3.740, *P* = 0.002), while the CE angle was 29 ± 5.9° in group 1A and 20 ± 6.3° in group 1B (*t* = − 3.172, *P* = 0.006) at the last follow-up. Regarding the limb-length discrepancy, group 1 and 2 were similar whereas group 1A and 1B were statistically different (group 1A: 0.7 ± 0.7 cm; group 2: 2.6 ± 1.0 cm; *t* = − 4.324, *P* = 0.001).

**Conclusions:**

Ilio-sacral resection without reconstruction removing more than one- third of the I-A distance leads to an impairement of the limb-length discrepancy and an increase of the defect of the acetabular coverage without altering the functional outcome. Nevertheless, iliosacral resection without reconstruction could serve as a viable treatment option for pelvic type I-IV tumors.

## Background

Primary pelvis bone tumors around the iliosacral joint are difficult to treat because of the large size of tumors, difficulties in limb salvage surgery, and high recurrence after surgery. Previously these tumors were often treated with hindquarter amputation. Nowadays, limb-preserving procedures have emerged as viable surgical modalities. However, whether reconstruction is more beneficial after iliosacral bone tumor resection remains controversial. [1, 2] Because of the relatively high rates of complications and recurrence, only few patients benefit from such reconstruction, although no reconstruction leads to superior and posterior migration of the residual bone, resulting in subluxation of the ipsilateral hip joint. [3] This study was conducted to evaluate the functional outcomes of patients after partial iliosacral resection, and to reveal the changes that may occur in the hip joint.

## Methods

From March 1998 to October 2016, 21 patients (9 males and 12 females, aged 20–66 years) with pelvic bone tumors around the iliosacral joint were treated with iliosacral resection in our department; 18 were treated without reconstruction and 3 underwent reconstruction with autografts. Criteria for inclusion were: (1) confirmed histologic diagnosis of the tumor; (2) treated with iliosacral resection, the ipsilateral hip joint being preserved; (3) follow-up of at least 1 year. Histologic diagnosis was verified by experienced pathologists with expertise in musculoskeletal oncology at our institution. Primary malignant bone tumors were found in 13 patients (6 with chondrosarcoma, 3 with Ewing sarcoma, 2 with osteosarcoma, 1 with undifferentiated pleomorphic sarcoma, 1 with fibrosarcoma); 6 patients had primary benign bone tumors (4 with giant cell tumors, 1 with desmoid tumor, 1 with chondromyxoid fibroma); and 2 patients had metastatic tumor (1 from prostate cancer and 1 from clear cell renal carcinoma).

Postoperative follow-up was conducted at regular intervals of 3 months for the first 2 years, every 6 months for the next 3 years, and once a year after 5 years. The follow-up time was defined as time elapsed from the operation date to the latest follow-up date.

Two evaluation methods, the Musculoskeletal Tumor Society (MSTS) 1993 [4] rating scale and the Toronto Extremity Salvage Score (TESS), [5] were used to evaluate the functional outcomes. The MSTS 1993 score includes pain, function, emotional acceptance, supports, walking ability, and gait, and ranges from 0 to 30; the final MSTS 1993 score is presented as a percentage. The TESS is a patient-based measure of physical disability developed specifically for patients with extremity sarcoma, which evaluates the patient’s perceptions in difficulty with activities of daily living, mobility, work, and recreation. The final TESS ranges from 0 to 100. Higher scores indicate a higher level of function for both measures.

**Fig. 1:**
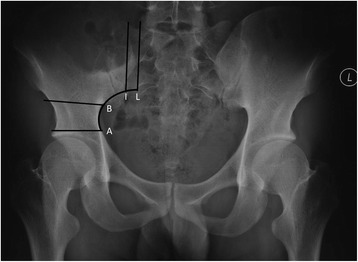
I-A distance means the line from the iliosacral joint(I) to the upper line of the acetabulum(A) along the curved line. Line L and line B are the osteotomy lines. Defect length was defined as the distance between the osteotomy lines along the curved line

**Fig. 2:**
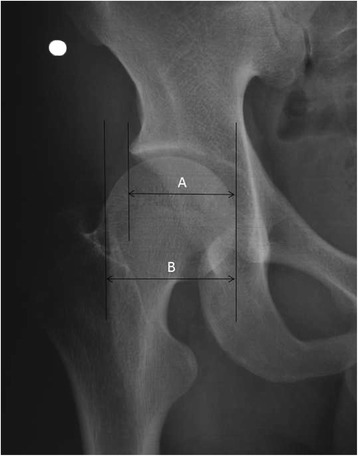
Acetabulum-head index(AHI). Line A is a horizontal measurement from the innermost surface of the head to a vertical line projected from the outermost margin of the acetabulum; line B is a similar horizontal measurement from the innermost surface of the head to a vertical line projected from the outermost surface of the head. The index is defined as A/B × 100

**Fig. 3:**
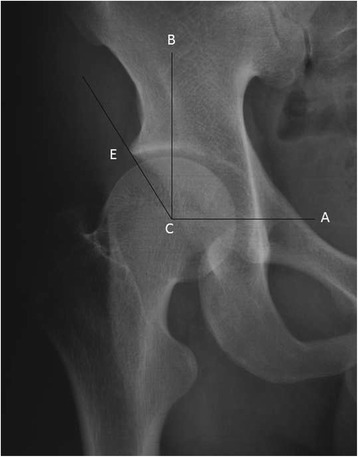
Center- edge angle. From the center (C) of the femoral head a line (C-A) is drawn through the center of the head of the opposite side. Perpendicularly to this line and through the center (C) the line (C-B) is raised. The CE angle denotes the angle between the line C-B and a line from C to the acetabular edge(E)

I-A distance was defined as the distance from the iliosacral joint to the upper line of the acetabulum along the curved line (Fig. 1), from which the percentage of defect after resection was calculated. Acetabulum-head index (AHI) initially described by Heyman et al.[6] (Fig. 2) and center-edge angle (CE angle) according to Wiberg et al.[7] (Fig. 3) were measured, and the relationships between defect percentage and femoral head coverage status were analyzed according to the X-ray findings.

Patients in group 1 did not undergo reconstruction after tumor resection, whereas those in group 2 underwent reconstruction with autografts. Patients in group 1 were further subdivided into two groups: group 1A included the patients with a defect less than one-third of the I-A distance and group 1B comprised the remainder.

The statistical analysis of the CE angle and AHI between groups was performed using the Statistical Program for the Social Sciences (SPSS, version 21.0). Independent sample t test was used to test the difference of CE angel and AHI between Group 1 and Group 2, and the difference between Group 1A and Group 1B. Statistical significance level set at *P* < 0.05.

## Results

The mean follow-up was 67.3 months (range, 14–163 months) (Table 1) for patients in group 1 (*n* = 18) and 27 months (range, 21–36 months) for those in group 2 (*n* = 3). There were 6 males and 12 females of average age 36.9 years (range, 20–66 years) in group 1, and 3 males of average age 28 years (range, 22–37 years) in group 2(*p* > 0.05). 3 patients with Ewing sarcoma and 2 with osteosarcoma received neoadjuvant with methotrexate(MTX), Adriamycin(ADM), cisplatinum(DDP) and ifosfamide(IFO). The mean surgical time was 194.4 min (range, 100–280 min) for the 18 patients in group 1 and 440 min (range, 210–720 min) for the 3 patients in group 2. The average blood loss was 1988 mL (range, 200–10,000 mL) in group 1 and 3266 mL (range, 1400–7000 mL) in group 2 (Table 2). Three patients (16.7%) who had recurrences after surgery (1 with chondrosarcoma, 1 with giant cell tumor, and 1 with desmoid tumor) underwent secondary local surgery, with no further recurrence or distant metastasis being observed at the latest follow-up. Two of three patients in group 2 achieved bone healing within 8 months of surgery. The autograft was absorbed in one patient, whereby the pelvis stabilized with the residual supra-acetabular bone rotating proximally and medially to lie against the sacrum, similar to the situation for patients in group 1 without reconstruction.

**Table 1:** 21 patients with pelvic tumors underwent iliosacral resection

Case	Group	Sex	Age	Diagnosis	Stage	Resection	outcome	Local relapse	Metastases	Follow-up (M)
1	1A	M	39	Metastatic tumor	–	Marginal	CDF	No	No	32
2	1A	F	66	Chondrosarcoma	IB	inter-lesional	CDF	No	No	72
3	1A	F	25	Desmoid tumor	3	inter-lesional	CDF	Y	No	59
4	1A	F	23	Giant cell tumors	3	inter-lesional	CDF	Y	No	48
5	1A	F	33	Giant cell tumors	3	inter-lesional	CDF	No	No	163
6	1A	F	45	Undifferentiated pleomorphic sarcoma	IIB	Wide	CDF	No	No	119
7	1A	F	34	Chondromyxoid fibroma	3	Wide	CDF	No	No	12
8	1A	F	23	Ewing sarcoma	IIB	Wide	CDF	No	No	26
9	1B	F	34	Chondrosarcoma	IB	inter-lesional	CDF	Y	No	104
10	1B	M	31	Chondrosarcoma	IB	Marginal	CDF	No	No	14
11	1B	M	51	Fibrosarcoma	IIB	Marginal	CDF	No	No	40
12	1B	M	20	Osteosarcoma	IIB	Wide	CDF	No	No	107
13	1B	M	37	Osteosarcoma	IIB	Wide	CDF	No	No	39
14	1B	F	31	Giant cell tumors	3	inter-lesional	CDF	No	No	114
15	1B	F	23	Giant cell tumors	3	inter-lesional	CDF	No	No	152
16	1B	F	23	Chondrosarco	IB	Wide	CDF	No	No	48
17	1B	M	61	Metastatic tumor	–	Wide	CDF	No	No	24
18	1B	F	65	Chondrosarco	IB	Marginal	CDF	No	No	17
19	2	M	22	Ewing sarcoma	IIB	Wide	CDF	No	No	21
20	2	M	25	Ewing sarcoma	IIB	Wide	CDF	No	No	24
21	2	M	37	Chondrosarco	IB	Marginal	CDF	No	No	36

*CDF* continously disease free

**Table 2:** Results of all the data measured in each group

	Groups1	Group2	*P*	Groups1A	Groups1B	*P*
Number(n)	18	3	–	8	10	–
Age	36.9(20–66)	28(22–37)	0.333	36.0(23–66)	37.6(20–65)	0.829
Bleeding(ml)	1988(200–10,000)	3266(1400–7000)	0.384	1400(200–3000)	2460(800–10,000)	0.316
Surgical time(min)	194(100–280)	440(210–720)	0.241	179(120–250)	206(100–280)	0.358
Pre-CE(°)	32(27–40)	29(27–33)	0.221	32(29–35)	32(27–40)	0.738
Post-CE(°)	24(10–35)	25(22–27)	0.849	29(21–35)	20(10–32)^a^	0.006
Change of CE(°)	7(−1–20)^a^	4(1-11)	0.430	3(− 1–9)	11(4–20)^a^	0.003
Pre-AHI(%)	82(79–86)	82(81–82)	0.406	84(80–86)	81(79–85)	0.190
Post-AHI(%)	73(50–88)	77(72–82)	0.485	80(72–88)	67(50–76)^a^	0.002
Change of AHI(%)	9(−2–30)^a^	4(− 1-10)	0.380	3(− 2–13)	14(6–30)^a^	0.006
MSTS(%)	93.3(80–100)	93.3(93.3–93.3)	0.950	94.3(90–96.7)	93(80–100)	0.602
TESS	97.8(90.4–100)	98.5(98.5–98.5)	0.770	98.9(97.8–100)	97.3(90.4–100)	0.347
LLD(cm)	1.7(0–5)	1.0(0–2)	0.373	0.7(0–2)	2.6(1–5)	0.001

^a^Except the patient who had a dislocation of the hip joint 60 months after operation. Pre:Preoperative,Post:Postoperative,CE:Center-edge angle, AHI:Acetabulum-head index,LLD:Limb-length discrepancy

The AHI and CE angle were measured to evaluate the changes in the ipsilateral hip joint. The preoperative AHI was not significantly different between group 1 (82 ± 2.4%) and group 2 (82 ± 0.6%, *t* = 0.855, *P* = 0.406), nor was the preoperative CE angle (group 1: 32 ± 3.3; group 2: 29 ± 3.2; *t* = 1.267, *P* = 0.221). Postoperatively, neither the AHI (group 1: 73 ± 10.1%; group 2: 77 ± 5.0%; *t* = − 0.713, *P* = 0.485) nor CE angle (group 1: 24 ± 7.7; group 2: 25 ± 2.6; *t* = − 0.193, *P* = 0.849) was significantly different. The preoperative AHI and CE angle were not significantly different between groups 1A (*n* = 8) and 1B (*n* = 10). One patient identified in group 1B developed a dislocation of the ispsilateral hip joint. Significant differences were found between groups 1A and 1B at the latest follow-up, at which time the AHI of was 80 ± 5.4% in group 1A and 67 ± 9.0% in group 1B (*t* = − 3.740, *P* = 0.002), while the CE angle was 29 ± 5.9 in group 1A and 20 ± 6.3 in group 1B (*t* = − 3.172, *P* = 0.006) (Table 2).. Both parameters associated with the percentage of defect from the iliosacral joint to the upper line of the acetabulum (Fig. 1, I-A distance). At the latest follow-up, 10 patients had AHI of less than 75%, 80% (8/10) of whom had defect percent I-A distance of more than one-third. The postoperative CE angle was less than 20° in 6 patients, all of whom had a defect percent I-A distance of more than one-third.

**Fig. 4:**
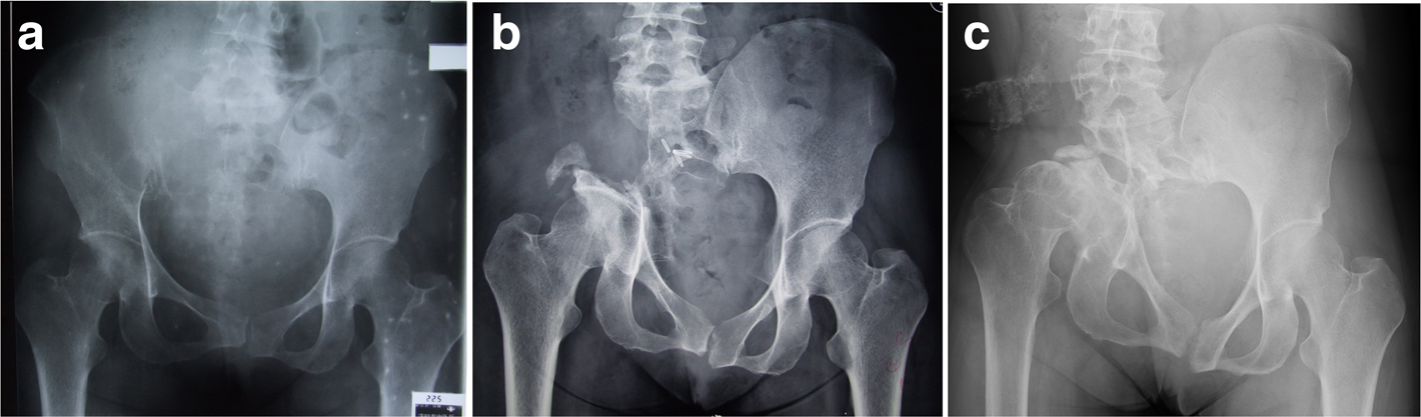
A 31 years old women diagnosed with GCT underwent iliosacral resection without reconstruction (**a**). She began to bear weight 2 months after surgery and had a good function after 3 months after surgery(MSTS 93 score was 90%) (**b**). But the poor situation of AHI and CE angle were progressing and a dislocation was found at the followup 60 months after surgery, though the function was good (MSTS 93 score was 93%) (**c**)

One patient in group 1B, a 31-year-old woman diagnosed with giant cell tumor of bone, developed a dislocation of the hip joint at the latest follow-up. She underwent iliosacral resection without reconstruction, comprising posterior osteotomy through the sacrum and another osteotomy through the supra-acetabular bone close to the acetabular dome. This patient began to bear weight 2 months after surgery and achieved good function 3 months after surgery (MSTS 93 score 90%). However, the poor status of AHI and CE angle continued to progress, and a dislocation was found at follow-up 60 months after surgery, although her function remained good (MSTS 93 score 93%) at the latest follow-up (Fig. 4).

No significant difference of limb-length discrepancy was observed between group 1(mean 1.7 cm; range 0–5 cm) and group 2(mean 1.0 cm; range 0–2 cm). However, the limb-length discrepancy of group 1A was significantly better than group 1B (group 1A: 0.7 ± 0.7 cm; group 1B: 2.6 ± 1.0 cm; *t* = − 4.324, *P* = 0.001). Eight of 10 patients in group 1B had more than 2 cm of discrepancy, while only 1 patient in group 1A and 1 patient in group 2 had a discrepancy of more than 2 cm.

**Fig. 5:**
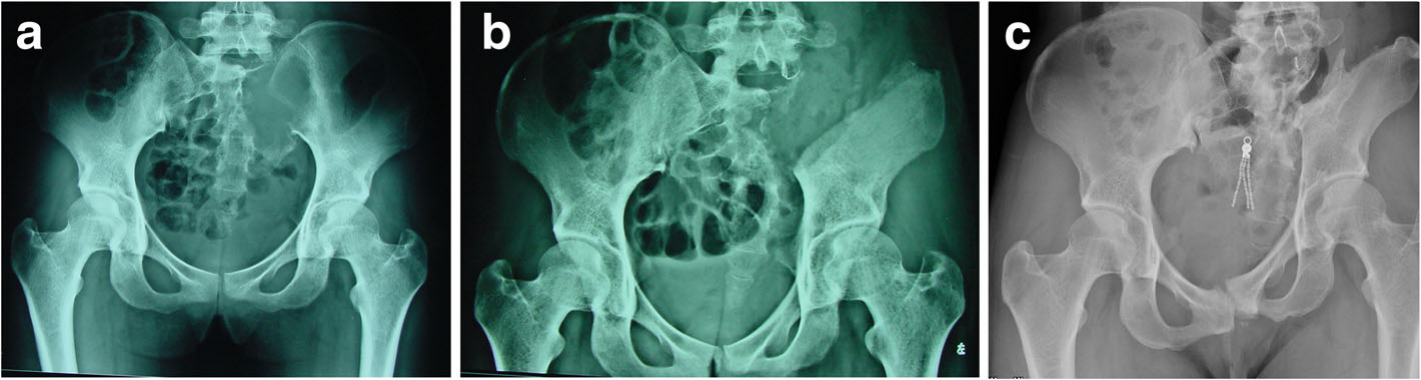
A 23-year-old woman with Giant cell tumor of the left iliosacral joint (**a, b**). Photographs obtained 135 months postoperatively. The preoperational CE angle and AHI was 33°and 83%, compare with 25° and 76% 135 months after surgery. The final MSTS functional score of the patient was 29/30, her gait was almost normal (**c**)

Functional outcome data were available for 14 of the 18 patients in group 1 and for 2 of the 3 patients in group 2. The final average MSTS 1993 score was 93.6% (range, 80–100%) in group 1 and 93.3%(range, 93.3–93.3%) in group 2. The mean TESS was 98 (range, 90–100) in group 1 and 98.5 in group 2 (Fig. 5). Both MSTS and TESS were not significantly different between group 1 and group 2. And also no significantly difference was observed between group 1A and group 1B (Table 2). All patients could walk without a walking aid, including the woman who developed a dislocation of the ipsilateral hip joint.

None of the 21 patients acquired deep wound infection. One patient in group 1 had intraoperative sacral nerve damage, which led to temporary incontinence. Two patients in group 1 contracted wound necrosis, one of whom was cured by irrigation and debridement; the other’s dressings were changed, leading to resolution after 2 months. The autograft was absorbed in one patient in group 2, whose screws were displaced 6 months after surgery.

## Discussion

Limb salvage surgery in pelvic bone tumors around the iliosacral joint is difficult. Since hindquarter amputation causes severe physical disabilities and poor quality of life, limb salvage surgery is worthwhile if it can achieve better functional outcomes and quality of life. However, the location, histologic diagnosis, size of the tumors, risk of local recurrence, and the estimated postoperative outcome should be taken into consideration when choosing between amputation and limb salvage surgery [1, 8].

As the ilium and sacrum become disconnected after resection of tumors, whether or not to reconstruct the pelvis is a controversial issue. Some researchers hold the view that reconstruction is necessary because it may stabilize the pelvic bones and preserve limb function, whereas others believe that reconstruction is unnecessary. Regardless of the method chosen to reconstruct, there are inherent difficulties in achieving sacroiliac arthrodesis. None of our patients had an iliosacral defect small enough to be closed primarily and wired to achieve direct bony healing. Fixation of the structural bone grafts is also problematic. The amount and quality of the sacrum available for screw fixation without damage to the sacral nerve roots was limited after resection. At the distal site, the osteotomy often was close to the acetabular dome, making stable screw placement problematic. Prolonged surgery time, difficulty with wound closure, and frequent complications also make successful bone graft reconstruction difficult.

Given that postoperative functional outcomes are the principal concern of clinicians, it is necessary to evaluate the functional outcomes when determining whether a reconstruction is to be conducted. There are few reports on functional outcomes after resection of bone tumors around the iliosacral joint. In a 10-case series by O’Connor et al., [9] after resection of the iliosacral joint 7 patients underwent iliosacral joint reconstruction, 5 of whom were rated “good” or “very good” on the MSTS rating scale while the remaining 2 were rated “bad.” In the other 3 patients who did not undergo reconstruction, 2 were rated “good” and 1 was rated “medium to good.” Gordon et al. [2] reported 16 patients who underwent resection of the iliosacral joint. The functional outcomes in this study were similar between the 12 patients who did not undergo reconstruction and the 4 who underwent reconstruction, with no significant difference. All 4 patients who underwent reconstruction needed walking aids, whereas among the 12 patients who did not undergo reconstruction, 9 were able to walk without aids. In the present study, the final average MSTS 1993 score was 93.6% (range, 80–100) in group 1 and 93.3% in group 2. The mean TESS was 98 (range, 90–100) in group 1 and 98.5 in group 2. All patients thus had good function that met the needs of daily life and work.

In the present study, both AHI and CE angle associated with the percentage defect of I-A distance. In group 1, patients with a defect greater than one-third the I-A distance had smaller CE angles and lower AHI than patients with defect less than one-third the I-A distance. One patient who underwent osteotomy very close to the acetabular dome developed a partial dislocation of the hip joint 60 months after surgery, which progressed until the latest follow-up, indicating that the larger gap between the neighboring remaining sacral bones caused rotation and upward shift of the remaining pelvis bones, thus worsening the femoral head coverage. Although only one case of this series developed dislocation, given the small sample size of group 1, the upper 95% confidence interval of having a dislocation is 17%, and even worse if only the 10 in group 1B are considered, is 33%. Considering that the average age was only 36.9 years old in this group and the short follow-up, whether the lack of coverage will progress and whether more patients will develop a dislocation in the future remain unknown. The lack of coverage likely creates a situation similar to that of a dysplastic acetabulum, potentially increasing the occurrence of hip arthritis. However, how many patients will be affected and when the arthritis will occur are uncertain. Nevertheless, the functional outcomes are good in this group. Even for the patient with partial dislocation of the hip joint, the MSTS score was 93% and only medium-level claudication was observed.

Complications are common in pelvis bone tumor surgeries. Researchers in the Rizzoli Institute followed up 270 patients who underwent pelvis bone tumor surgery and reported that infection emerged in 15% of patients among 133 non-reconstruction patients and in 26% of patients among 137 who underwent reconstruction. The risk of infection was apparently higher in reconstruction patients.[3] Gordon et al. [2] reported that 3 of 4 patients who underwent reconstruction suffered wound complications requiring repeated resection, whereas among 12 patients who did not undergo reconstruction, wound complications emerged only in 4 patients. The risk of complications was lower in patients who did not undergo reconstruction. In other reports, the overall recurrence rate is 14–16%. In the present study, 3 patients in group 1 suffered complications. One patient had intraoperative sacral nerve damage that led to incontinence. Two patients developed wound necrosis, one of whom was treated by irrigation, debridement, and wound reclosure, while the other received a change of dressing and was cured after 2 months. No patients acquired deep wound infection. One patient developed deep venous thrombosis of the lower limb and was treated with immobilization and anticoagulant therapy, and no subsequent complications were observed. No pulmonary embolism or other severe postoperative complications were apparent. The autograft was absorbed in one patient in group 2, whose screws were displaced 6 months after surgery.

There are some limitations to our study. Firstly, as we were able to evaluate the situation using only anteroposterior X-ray films, the elaborate 3-D structure of the pelvis bones could not be analyzed. Secondly, our sample size is small, which somewhat weakens the robustness of our statistical analysis. Thirdly, only patients older than 15 years are included in this study, which thus lacks outcome data for younger patients. Lastly, the follow-up is relatively short; therefore, the number of patients who may develop a dislocated hip joint and develop arthritis in future is uncertain.

Based on the reasonable function, fewer complications, shorter surgery time, less blood loss, and lack of straightforward reconstructive options, iliosacral resection without reconstruction could serve as a viable treatment option for pelvic type I–IV tumors. The AHI and CE angle associated with the defect percentage from the iliosacral joint to the upper line of the acetabulum. Whether or not the poor femoral head coverage will increase the occurrence of hip arthritis remains unknown and will need a longer follow-up of a larger cohort.
